# Are publicly available internet resources enabling women to make informed fertility preservation decisions before starting cancer treatment: an environmental scan?

**DOI:** 10.1186/s12911-018-0698-3

**Published:** 2018-11-19

**Authors:** N. Mahmoodi, H. L. Bekker, N. V. King, J. Hughes, G. L. Jones, on behalf of the Cancer, Fertility and Me research team, Galina Velikova, Diana Greenfield, Robert Phillips, Sheila Lane, Greta Brauten-Smith, Jacqui Gath, John Snowden, Daniel Yeomanson, Ellissa Baskind, Tonia Campbell, Frances Darby, Katharina Vogt, Jonathan Skull, Daniel Stark

**Affiliations:** 1 Department of Psychology, School of Social Sciences, Leeds Beckett University, Leeds, LS1 3HE UK; 2 Institute of Health Sciences - School of Medicine, University of Leeds, Leeds, UK; 3 School of Health & Related Research, University of Sheffield, Sheffield, UK

**Keywords:** Patient decision aid, Shared decision making, Fertility preservation, Cancer treatment, eHealth, Environmental scan

## Abstract

**Background:**

To identify publicly available internet resources and assess their likelihood to support women making informed decisions about, and between, fertility preservation procedures before starting their cancer treatment.

**Methods:**

A survey of publically available internet resources utilising an environmental scan method. Inclusion criteria were applied to hits from searches of three data sources (November 2015; repeated June 2017): Google (Chrome) for patient resources; repositories for clinical guidelines and projects; distribution email lists to contact patient decision aid experts. The Data Extraction Sheet applied to eligible resources elicited: resource characteristics; informed and shared decision making components; engagement health services.

**Results:**

Four thousand eight hundred fifty one records were identified; 24 patient resources and 0 clinical guidelines met scan inclusion criteria. Most resources aimed to inform women with cancer about fertility preservation procedures and infertility treatment options, but not decision making between options. There was a lack of consistency about how health conditions, decision problems and treatment options were described, and resources were difficult to understand.

**Conclusions:**

Unless developed as part of a patient decision aid project, resources did not include components to support proactively women’s fertility preservation decisions. Current guidelines help people deliver information relevant to treatment options within a single disease pathway; we identified five additional components for patient decision aid checklists to support more effectively people’s treatment decision making across health pathways, linking current with future health problems.

**Electronic supplementary material:**

The online version of this article (10.1186/s12911-018-0698-3) contains supplementary material, which is available to authorized users.

## Background

Providing accurate patient information is fundamental to health services worldwide [1]. Since the 1940s, social science research has informed guidance to enhance text readability [2, 3] and health communications [4, 5]. National and academic organisations provide best practice guidance [6] for patient-focused intervention development and evaluation [7, 8]. Research indicates patient resources informed by these standards enhance health literacy and patient benefits [9]. A challenge for service delivery is to support patient-focused communications about complex health problems; most guidance support one-off decisions about a health problem in a single pathway of care [10].

An iatrogenic consequence of cancer treatment is an increased chance of impaired fertility; treatments can permanently damage the endocrine function and/or reproductive systems needed to fall pregnant or carry a baby to term [11]. Oncologists deliver care to minimise these fertility-related effects using minimally gonadotoxic therapies [12] and/or fertility sparing procedures (e.g. trachelectomy, ovarian transpositioning, shielding) [13, 14]. For some women with impaired fertility after cancer treatment, infertility treatments are offered [15]. Having fertility preservation procedures before cancer treatment may increase the likelihood of women having genetically related children in the future, but can delay the start of cancer treatment by a few weeks. Fertility services offer the following preservation procedures: embryo cryopreservation, oocyte cryopreservation, and ovarian tissue cryopreservation [16, 17].

Integrating relevant fertility preservation information into cancer pathways is essential for women to make informed decisions about whether to undergo fertility preservation, and/or which procedures to choose [15, 18–25]. Receiving accurate and timely information is associated with reported better quality of life and reduced decisional regret, post cancer treatment [22, 26–29]. However, women’s recall of discussions with health professionals and support about infertility-related side-effects of cancer treatment is low [18, 30, 31]; findings indicate variation in the timing, content, utility and quantity of information about fertility preservation provided by cancer services [18, 31–33].

Increasingly the internet is accessed for health information [34–38], and people find resources from stakeholders independent of healthcare services (e.g. public, professionals, charities, advocacy groups, product advertisers, unregulated businesses) and in varied formats (e.g. audio, video, text). These resources may provide accurate information to support patient and carer health literacy, or they may be misleading and difficult to understand in the context of a person’s life, experience and illness. It is unclear if and how systematically best practice guidance are used to inform publically available resources [39, 40]. This paper investigates what publically available resources are available to women about fertility preservation choices before starting their cancer treatment, and if they are sufficient to enable informed decision making.

## Methods

We carried out a survey of publically available patient information and guideline resources to support women diagnosed with cancer making fertility preservation choices before treatment. We employed an environmental scan method used in applied health research for systematic analysis of Google search engine, targeted website searches and contacting experts [41–44]. We used this approach to search for resources freely available to any woman or health professional [34–38, 44]; internet searches can be more effective at identifying reports [42] and informal material relevant to the topic than academic or organisational databases [45, 46].

We followed the PRISMA reporting guidelines for best practice in reporting systematic reviews of secondary data synthesis [47]. These guidelines provide steps to encourage methodological rigour around the search, inclusion criteria, extraction and synthesis of findings. The target for this environmental scan is a resource (leaflet or guideline) rather than an empirical study; the quality of the target is judged against criteria known to boost reasoning rather than those known to enhance methodological rigour. A data extraction sheet was developed to elicit systematically the key characteristics and content from each resource, which are described in tables and synthesised within the results section.

### Study context

This survey was carried out during the development phase of the Cancer, Fertility and Me Patient Decision Aid (*CFM-PtDA*) [48–50]. Other activities included (Nov 2015-Sept 2016): research governance and ethics; scoping local and national patient information provided during usual care by services to patients with cancer; mapping care pathways between cancer and fertility services in Leeds and Sheffield, UK; alpha testing [51] the CFM-PtDA prototype with patients, oncology healthcare professionals and other key stakeholders using qualitative methods. The project received ethics approval by National Health Service (NHS) Health Research Authority (HRA), East Midlands Nottingham 1 Research Ethics Committee in 2016, Ref: 16/EM/0122; HRA Ref:194751.

### Information sources and search strategies

Health information is provided and used for different purposes, such as to inform, reassure, persuade, acquire skills and enhance reasoning [52–54]. We searched the internet for resources with minimum standards for patient decision aids known to support understanding of the health problem [4, 55, 56]; provide awareness of the advantages and disadvantages of all relevant treatment options and their consequences [52]; and support reasoned decision making [17, 52–54]. These include components known to minimise bias through providing balanced, neutral information of all options and presentation of risk as natural frequencies/ percentages [57–61]; a visual representation of the decision problem [10, 62]; an evaluation of these details in accordance with a person’s values [63–66]; an explanation of people’s understanding of illness and treatment [4, 55, 56, 67]; and allow for an informed decision to be reached and implemented with health professionals [59, 63, 68].

Three types of data source were searched between November 11th – December 17th 2015, and repeated in June 2017. Search terms, Uniform Resource Locator (URL) identifiers, and hits were managed using Excel [42]:

1. Google (Chrome) was searched using 8 unique search themes (cancer, breast cancer, leukemia, lymphoma, gynaecological, surgery, radiotherapy, chemotherapy), developed with an information specialist (NVK). Within those themes there were 9 unique strategies which contained multiple combinations of the following terms (UK and USA spellings): cancer (all types); women (patient); treatment (procedure); fertility preservation (treatment); decision-making; information (booklet, education, decision aid) (contact authors for further information). All internet web-links, including sponsored links, on the first 5 pages of each search were screened (*n* = 3600 websites) [42].
2. Open access repositories of patient decision aids, clinical guidelines and active research were searched using five search strategies developed with an information specialist (NVK): Decision Aids Library Inventory (DALI, Ottawa Health Research Institute, Canada) (https://decisionaid.ohri.ca/cochinvent.php); Trip clinical search engine https://www.tripdatabase.com/); Clinical guidelines database- NICE Evidence; (https://www.evidence.nhs.uk/); Agency for Healthcare Research and Quality National Guidelines Clearinghouse - AHRQ-NGC; (https://www.guideline.gov/); UK and USA Clinical trials databases (https://www.ukctg.nihr.ac.uk/; https://clinicaltrials.gov/).
3. Experts (health professionals, patients and researchers) in patient decision aid and shared decision-making interventions and research were contacted via the SHARED-L international email distribution list. Experts were not asked to participate in the study, but to simply email the study team with information on any patient resources and guidelines which met the study criteria. Four experts responded by 31st January 2016, and all open-access resources identified (*n* = 4) were included in the study.

### Data selection

Patient resources with the following criteria were included:

- Targeted women diagnosed with cancer and offered fertility preservation options,
- Described fertility problems as a consequence of cancer treatment,
- Described fertility preservation options and consequences
- Contained an explicit statement to consider fertility preservation options before cancer treatment.

Clinical guidelines with the following criteria were included:

- Raised awareness of the link between cancer treatment and fertility problems
- Contained explicit guidance on what fertility preservation options to mention to women diagnosed with cancer
- Explained the links between the fertility and cancer management pathways
- Provided explicit guidance on how to support women’s choices about fertility preservation options in the context of their cancer care.

All resources were screened (NM) for inclusion in the study; NM and HLB discussed decisions about resources, included (*n* = 10), excluded (n = 10) and uncertain (all). The search process and criteria were discussed independently with GLJ, JH (November 2015).

### Data extraction

A data extraction sheet (Additional file 1) was developed (HLB, NM) with reference to patient decision aid research reviews and resource development [2, 7, 8, 10, 35, 52–54, 69, 70], and cancer-related fertility preservation decision aids [22, 26, 27, 29, 71, 72]. The presence or absence of minimum standards and components know to support understanding of the health condition, decision problem, treatment options and their consequences, and reasoned decision making were extracted systematically from each resource meeting the scan’s inclusion criteria using the data extraction sheet. The Data Extraction Sheet was piloted (NM, JH, GLJ), independently reviewed by the *CFM-PtDA* project steering group (February 2016), and applied systematically to eligible resources (NM, JH) extracting the following:

- Characteristics: type of ‘e-resource’ (internet-delivered, internet-adapted, internet-available) [35], title, publisher, year of publication and of the updated year, country, authors, funders, location (URL), length, stated purpose resource.
- Quality indicators: Flesch readability formula [3] was used to measure comprehensibility of leaflet (70–79 fairly easy; 60–69 standard; 50–59 fairly difficult; 30–50 difficult; 0–29 confusing), endorsed by third party, developed systematically, listed evidence used to inform content.
- Describes health problem: label and symptoms, cause, time-line, consequences, cure and/or control, and emotional responses to a) cancer, b) fertility / infertility, and c) cancer-related infertility.
- Describes treatments: label /procedure, eligibility, prognosis, side effects short term, side effects long term for cancer treatment (chemo/ radio/ hormone therapy, surgery), fertility preservation treatments (egg/ embryo/ ovarian freezing, ovarian suppression/ shielding), and/or infertility treatments (in vitro fertilisation, adoption/ fostering, surrogacy).
- Signposts illness-wellness trajectory: care pathways; quality of life.
- Decision architecture to boost/ bias thinking: trade-offs between options; decision picture; decision guidance; other’s values/ stories; risk presentation; treatment preference.
- Health service engagement: prompts to prepare for consultations, friends and families; diagrams/ guidance to prepare for procedures; signposting to other information.

### Data synthesis and analysis

Two quality-assessment grids were applied to synthesise evidence across resources: The IPDAS grid - 12 components identified as minimum criteria for a patient decision aid resources [8, 9, 44, 69, 70]; Informed Decision-Making (IDM) grid - 10 components known to boost informed and shared decision-making, and minimise reasoning bias [52–54]. Each item scored either 0 (present) or 1 (not present); Summed total scores were IPDAS grid (0–12), and IDM grid (0–10).

The findings are presented using narratives and frequency statements to address whether or not women and health professionals have access to publicly available online resources that support fertility preservation decisions before cancer treatment. SPSS statistical software was used to manage the data elicited from the resources. Descriptive data were used to assimilate findings across resources, and show the number of occurrences for each component on the data extraction sheet.

## Results

**Fig. 1:**
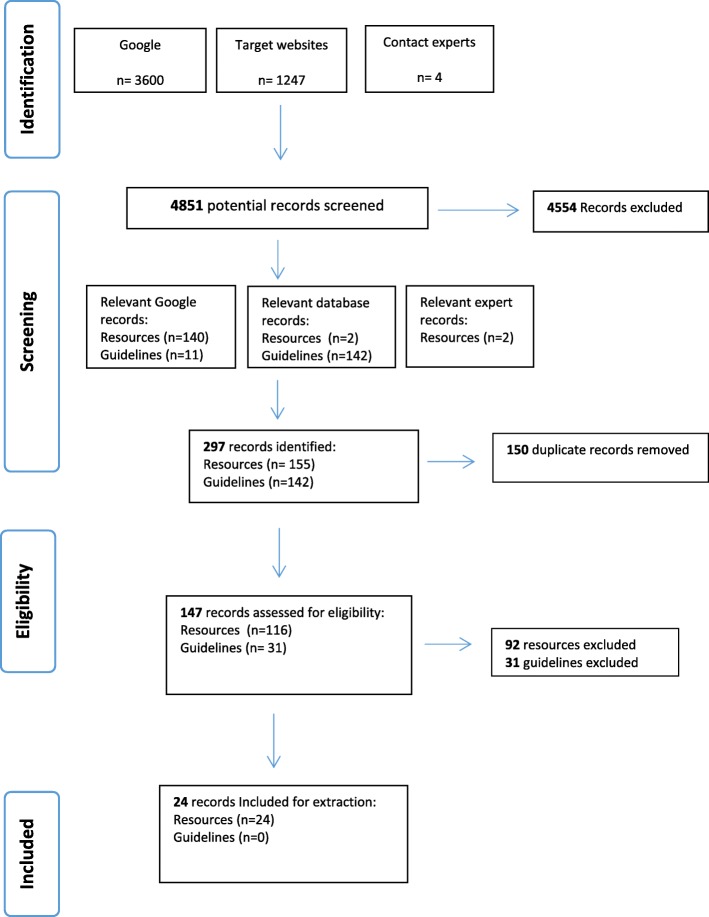
Study flow diagram - Resource and guideline identification, screening, and eligibility

Shown in Fig. 1, the search yielded 147 unique records eligible for assessment (*n* = 116 e-resources and *n* = 31 guidelines). Following screening, none of the 31 guidelines met the scan’s inclusion criteria (Table 1); although all guidelines make links between the consequences of cancer treatment and fertility problems, only 7 highlight guidance around availability of fertility preservation treatments, only 5 made links between cancer and fertility management pathways for women, and none provided guidance for professionals on how to support women’s decision making about fertility preservation options in the context of their cancer care.

**Table 1:** Characteristics of guidelines meeting eligibility criteria (*n* = 0)

Guideline title	Organisation (Country)	Year of publication	Year guideline identified	Eligibility criteria 1^a^	Eligibility criteria 2^a^	Eligibility criteria 3^a^	Eligibility criteria 4^a^
Fertility preservation for AYAs diagnosed with cancer: Guidance for health professionals	The clinical practice guideline portal (Australia and New Zealand)	2011	2015 & 2017	x			
South Australian Gynaecological Cancer Care Pathway Optimising Outcomes for Women with Gynaecological Cancer	The clinical practice guideline portal (Australia and New Zealand)	2014	2015 & 2017	x			
Clinical practice guidelines for the management and support of younger women with breast cancer	National health and medical research council (Australia and New Zealand)	2004	2015 & 2017	x			
Clinical practice guidelines for the management of women with epithelial ovarian cancer	National health and medical research council (Australia and New Zealand)	2004	2015 & 2017	x			
Clinical practice guidelines for the psychosocial care of adults with cancer	National health and medical research council (Australia and New Zealand)	2003	2015 & 2017	x			
For the prevention, early Detection and management of colorectal cancer	National health and medical research council (Australia and New Zealand)	2005	2015 & 2017	x			
The effects of cancer treatment on reproductive functions. Guidance on management	The Royal College of Physicians (UK)	2007	2015 & 2017	x			
Fertility Sparing Treatments in Gynaecological Cancers	Royal College of Obstetricians and Gynaecologists (UK)	2013	2015 & 2017	x			
Pregnancy and Breast Cancer	Royal College of Obstetricians and Gynaecologists (UK)	2011	2015 & 2017	x			
Long term follow up of survivors of childhood cancer	SIGN (UK)	2013	2015 & 2017	x	x		
Management of epithelial ovarian cancer	SIGN (UK)	2013	2015 & 2017	x	x		
Management of cervical cancer	SIGN (UK)	2008	2015 & 2017	x			
Gynaecological cancer: guidance for nursing staff	Royal College of Nursing (UK)	2005	2015 & 2017	x			
Classical Hodgkin’s Lymphoma; 1st Line	British Committee for Standards in Haematology (UK)	2014	2015 & 2017	x	x		
For the diagnosis and Management of lymphoma	National Health and Medical Research Council (UK)	2005	2015 & 2017	x	x		
Fertility problems and assessment	NICE (UK)	2014	2015 & 2017	x		x	
Fertility Pathway	NICE (UK)	2014	2015 & 2017	x	x	x	
Guidelines for the investigation and management of nodular lymphocyte predominant Hodgkin lymphoma	British Committee for Standards in Haematology (UK)	2015	2015 & 2017	x			
Cancer, fertility and pregnancy	ESMO (European)	2010	2015 & 2017	x			
Cervical Cancer: ESMO Clinical Practice Guidelines for diagnosis, treatment and follow-up	ESMO (European)	2012	2015 & 2017	x			
Non-epithelial ovarian cancer: ESMO Clinical Practice Guidelines for diagnosis, treatment and follow-up	ESMO (European)	2012	2015 & 2017	x			
Primary breast cancer: ESMO Clinical Practice Guidelines for diagnosis, treatment and follow-up	ESMO (European)	2015	2015 & 2017	x		x	
Preservation of Fertility in Paediatric and Adolescent Patients With Cancer	American Academy of Paediatrics (USA)	2009	2015 & 2017	x	x		
ACR Appropriateness Criteria pre-treatment planning of invasive cancer of the cervix	American College of Radiology - Medical Specialty Society (USA)	2011	2015 & 2017	x			
American Society of Clinical Oncology Recommendations on Fertility Preservation in Cancer Patients	American Society of Clinical Oncology (USA)	2006	2015 & 2017	x	x	x	
Cancer of the uterine cervix	Alberta health (USA)	2014	2015 & 2017	x			
Epithelial ovarian, fallopian tube, and primary peritoneal cancer	Alberta health (USA)	2012	2015 & 2017	x			
Ovarian germ cell tumours	Alberta health (USA)	2013	2015 & 2017	x			
Lymphoma	Alberta health (USA)	2014	2015 & 2017	x			
Management of gynaecologic issues in women with breast cancer	American College of Obstetricians and Gynecologists (USA)	2013	2015 & 2017	x			
Breast Cancer: Management and Follow-up	Clinical Practice Guidelines and Protocols in British Columbia (Canada)	2013	2015 & 2017	x		x	

^a^Eligibility criteria 1: Raised awareness of the link between cancer treatment and fertility problems

2: Contained explicit guidance on what fertility preservation options to mention to women diagnosed with cancer

3: Explained the links between the fertility and cancer management pathways

4: Provided explicit guidance on how to support women’s choices about fertility preservation options in the context of their cancer care

Following screening, 24 patient resources met the scan’s inclusion criteria (Table 2). Most resources were suitable for women with any cancer type (*n* = 16), and most were designed for adults (n = 16). Resources were published between 2005 and 2017, all but one (SN22) in English, one (SN24) was judged as fairly easy to read, and one (SN22) was internet-adapted by using an interactive web-based platform (Table 2). All resources stated their publisher, thirteen described the development team, ten included references of the evidence-base informing the resource content, four stated they were endorsed by a third party, three published peer-reviewed papers demonstrating the resource’s development and/or evaluation, and two were located on the publicly available DALI (Table 3).

**Table 2:** Characteristics of resources meeting eligibility criteria (n = 24)

Study No.	Source	Resource Title	Organisation (Country)	Resource Type^a^	Target Group	Cancer Type	Year resource identified	Year of publication (updated)	No. pages	Readability Flesch Score [2]
1	Google	Cancer Treatment Fertility- Information for Women	Macmillan Cancer Support (UK)	Internet- available	Adult	Any cancer	2015 & 2017	2013 (2016)	50	55
2	Google	Fertility care for Women Diagnosed with Cancer	Central Manchester University Hospital- St Mary’s Hospital (UK)	Internet- available	Adult	Any cancer	2015 & 2017	2013	16	48
3	Google	Save My Fertility: Fertility Preservation for Women Diagnosed with Cancer	The Onco-fertility Consortium (USA)	Internet- delivered, Internet- available & Mobile App	Adult & TYA	Any cancer	2015 &2017	2011	5	35
4	Google	Fertility and Breast Cancer Treatments	Breast Cancer Care (UK)	Internet- available	Adult	Breast	2015 &2017	2014 (2017)	22	52
5	Google	Fertility and Women with Cancer	American Cancer Society (USA)	Internet- delivered & Internet- available	Adult & TYA	Any cancer	2016 & 2017	2013	25	48
6	Google	Fertility and Pregnancy Issues During and After Breast Cancer	Breast Cancer.org (USA)	Internet- delivered	Adult	Breast	2015 & 2017	2015	21	57
7	Google	Fertility and Cancer: A Guild for People With Cancer, Their Friends and Families	Cancer Council (Australia)	Internet- available	Adult & TYA	Any cancer	2015 & 2017	2014 (2016)	86	45
8	Google	Breast Cancer and Fertility	Europe Donna Ireland (Ireland)	Internet- available	Adult	Breast	2015 & 2017	2008	16	48
9	Google	Oncofertility	Flinders Fertility (Australia)	Internet- available	Adult	Any cancer	2015 & 2017	2013	14	32
10	Google	Can I Still Have Children?	Melbourne IVF and Royal Women’s Hospital (Australia)	Internet- available	Adult	Any cancer	2015 & 2107	2013	20	41
11	Google	Fertility Concerns and Preservation for Women	Cancer.net (USA)	Internet- delivered	Adult	Any cancer	2015 & 2017	2014	5	27
12	Google	Fertility Preservation: Options for Women Who Are Starting Cancer Treatment	Memorial Sloan- Kettering Cancer Centre (USA)	Internet- available	Adult	Any cancer	2015 & 2017	2015	14	47
13	Google	Fertility	New Zealand Breast Cancer Foundation (New Zealand)	Internet- delivered	Adult	Breast	2015 & 2017	2013	2	47
14	Google	Caring for Adolescents and Young Adults	NCCN guidelines for Patients(USA)	Internet- available & Internet- delivered	TYA & Parents	Any cancer	2015 & 2017	2013	118	53
15	Google	Fertility Preservation in Cervical Cancer	North London Gynaecological Cancer Network (UK)	Internet- available	Adult	Gynaecological cancers	2015 & 2017	2015	10	42
16	Google	Fertility and Cancer	New life (UK)	Internet- delivered	Adult	Any cancer	2015 & 2017	2015	7	55
17	Google	Fertility Preservation for Women with Cancer: FAQs	Walgreens (USA)	Internet- available	Adult	Any cancer	2015 & 2017	2011	8	41
18	Google	Fertility Options to Consider Before Treatment Begins and Parenthood Options After Cancer	Fertile Hope & Cleveland Clinic (USA)	Internet- available	Adult	Any cancer	2015 & 2017	2005	12	49
19	Google	Fertility Preservation	University of Iowa Hospitals and Clinics (USA)	Internet- available	Adult	Any cancer	2015 & 2017	2013	21	48
20	Google	Future Fertility: Preserving Fertility in Women with Cancer	University of New South Wales, Onco-fertility Consortium, Prince of Wales Hospital (Australia)	Internet- delivered & Internet- available	Adult	Any cancer	2015 & 2017	2015	52	58
21	Google	Fertility Facts	Lymphoma and Leukaemia Society (USA)	Internet- available	TYA	Lymphoma and Leukaemia	2015 & 2017	2015	7	41
22	DALI database	Breast cancer and having children	Leiden University Medical Centre/ Pink Ribbon (Dutch)	Internet- adapted	Adult	Breast	2015 & 2017	2012	31	47
23	Expert contacts	Learning About Cancer and Fertility: A Guide for Parents of Young Girls	North western University/ Onco-fertility Consortium (USA)	Internet- available	Parents	Any cancer	2015 & 2017	2011	24	55
24	Expert contacts	Fertility-related choices: A decision aid for younger women with breast cancer	University of Melbourne/ Breast Cancer Network/ Prince of Wales Hospital (Australia)	Internet- available	Adult	Breast	2015 & 2017	2013 (2016)	57	78

^**a**^Resource type [35]: Internet-delivered include all PtDAs for which some or all parts are delivered using the Internet

Internet- available include PtDAs that were initially developed and tested in other formats (e.g., paper, audio, or video), then made available on the Internet for individuals to download, print, and complete

Internet- adapted include PtDAs created in other formats that were purposefully adapted to allow individuals to use them directly on the Internet. Examples include adapting paper worksheets into interactive questionnaires, and adapting text and video components of PtDA DVDs into websites

**Table 3:** Assessment of Resource Development Quality (*n* = 24)

Development Process Item	Study Number of Resource																								Number of resources including component
	1	2	3	4	5	6	7	8	9	10	11	12	13	14	15	16	17	18	19	20	21	22	23	24	
Publishers																									
Service providers		x							x	x		x			x			x	x						7
Charity organisation	x			x	x	x	x	x			x		x								x				9
Mixed (service, charity, academic)			x											x						x		x	x	x	6
Pharmaceutical companies																x	x								2
Stakeholders																									
Communication or decision scientist																						x		x	2
Charity representative			x		x		x							x										x	5
Patient or advocacy groups	x		x	x			x							x											5
Fertility professional					x	x	x	x									x		x			x		x	8
Cancer professionals	x		x	x	x	x	x	x						x								x		x	10
Primary healthcare professionals	x			x		x		x						x								x		x	7
Pharmaceutical organisation														x											1
Applied researchers						x	x															x		x	4
Health care professional organisation	x		x		x					x				x					x						6
Endorsement Referenced In Resource																									
Information standard	x			x																					2
Professional body																									0
Patient advocacy		x				x																			2
IPDAS endorsement																									0
Evidence-Base Referenced In Resource																									
Evidence-based publication in resource	x		x	x	x				x					x			x	x			x	x			10
Quality Publication About Resource																									
Publication of development/ evaluation			x																			x		x	3
Located on recognised repository (DALI)																						x	x	x	3

### Meeting minimum standards for patient decision aid resources

All resources eligible for the study provided information about the health conditions and treatment options (Table 4). The stated aim for 18 resources was to provide information about cancer, fertility and infertility options; 7 stated their purpose was to support making a decision between treatment options (SN8, SN10, SN11, SN12, SN18, SN22, SN24). Of these, 3 focused on decisions between having cancer treatment with or without fertility preservation (SN2, SN3, SN23), 3 between fertility preservation options (SN22, SN23, SN24), and 2 between infertility treatments to have a family (SN22, SN24).

**Table 4:** Resource assessed for inclusion of components within Patient Decision Aid Resources (IPDAS) (n = 24) [8, 69, 70]

IPDAS Checklist Item	Study Number of Resources Including Component																								Number of resources including component
	1	2	3	4	5	6	7	8	9	10	11	12	13	14	15	16	17	18	19	20	21	22	23	24	
1. Describes health condition for index decision	x	x	x	x	x	x	x	x	x	x	x	x	x	x	x	x	x	x	x	x	x	x	x	x	24
2. Explicitly describes the index decision being considered		x	x												x							x	x	x	6
3. Describes the options	x	x	x	x	x	x	x	x	x	x	x	x	x	x	x	x	x	x	x	x	x	x	x	x	24
4. Describes positive features all options																	x		x					x	3
5. Describes negative features all options		x		x	x							x					x		x			x		x	8
6. Describes what it is like to experience the psychosocial consequences of options																									0
7. Shows negative and positive features of all options in equal detail (text amount, equal stats/ consequences)																	x						x		2
8 Cites evidence used or links to document	x		x	x	x				x					x			x	x			x	x			10
9. Provides a publication date resource	x	x		x	x		x	x		x		x		x			x	x		x		x		x	14
10. Provides information about an update policy		x		x	x	x		x		x	x			x								x		x	10
11. Provides information about uncertainty level around event /outcome probabilities																									0
12. Provides information about the funding source used for development	x		x				x							x								x	x	x	7
Total score IPDAS (out of 12)	5	6	5	6	6	3	4	4	3	4	3	4	2	6	3	2	7	4	4	3	2	8	5	8	

All 24 resources encouraged women to talk to their cancer care and/or fertility specialist teams, and/or speak with friends and family; 15 (SN3, SN6, SN7, SN8, SN11, SN13, SN14, SN16, SN18, SN19, SN20, SN21, SN22, SN23, SN24) provided questions to support shared decision making in consultations with health professionals. No resources included all the components identified as part of the minimum standards for a patient decision aid [9]; the median score was 4 out of 12 points (range 2–8) (Table 4).

### Inclusion of components boosting or biasing informed decision-making

There were variations in how cancer and fertility problems were described across resources (Table 5), with gaps in details to help women’s understanding of the causal links between having cancer treatment and an increased likelihood of having fertility problems in the future. Most resources (*n* = 21) described fertility-related options before, during and after cancer. Four (SN14, SN22, SN23, SN24) resources used flow diagrams to illustrate links between choices and service delivery pathways. Seven provided diagrams and pictures to explain procedures (e.g. In-vitro fertilisation) (SN1, SN2, SN7, SN10, SN12, SN23, SN24) and six illustrated body systems (e.g. reproductive system) (SN1, SN7, SN10, SN11, SN12, SN23).

**Table 5:** Details of health conditions described, by illness-schemata category (n = 24) [56]

	Cancer (16/24)	Fertility (14/24)	Infertility (13/24)	Cancer-Related Infertility (24/24)
Label/symptom	3 (13%)	7 (29%)	4 (17%)	9 (38%)
Timeline	1 (4%)	6 (25%)	5 (21%)	21 (88%)
Cause	2 (8%)	11 (46%)	2 (8%)	14 (58%)
Consequence	15 (63%)	3 (13%)	5 (21%)	19 (79%)
Cure/control	5 (21%)	1 (4%)	7 (29%)	17 (71%)

There was variation in the amount of information given about treatment options for the short and long-term consequences of cancer-related fertility (Table 6). Ten resources included prompts encouraging women to describe what was important to them about infertility-treatment options (Table 7); one provided quality of life statements to help women’s reasoning (SN7). Three resources used an option-by-attribute table format to summarise details (SN5, SN23, SN24), and two used trade-offs based on their values (SN22, SN24). The health professionals’ opinion was provided in two resources (SN1, SN6), and other women’s stories about their experiences in four resources (SN1, SN7, SN14, SN24).

**Table 6:** Treatments described across resources for cancer and fertility problems (*n* = 24)

Cancer Treatment		Fertility Preservation Options	
Chemotherapy	23 (96%)	Egg freezing	22 (92%)
Radiotherapy	23 (96%)	Embryo freezing	23 (96%)
Surgery	17 (71%)	Ovarian tissue freezing	21 (88%)
Hormone therapy	8 (33%)	Ovarian suppression	13 (54%)
Targeted therapy	4 (17%)		
Ovarian suppression as part of cancer treatment	5 (21%)		
Family Planning During Cancer		Infertility Treatment/ Family Planning After Cancer	
Contraception	11 (46%)	Natural	17 (71%)
		Assisted conception	17 (71%)
		Surrogacy	16 (67%)
		Adoption / fostering	15 (63%)
		Contraception	12 (50%)

**Table 7:** Resource assessed for components boosting or biasing informed and shared decision making (I/SDM) (n = 24) [52–54]

I/SDM Component	Study Number of Resources including Component																								Number of resources including component
	1	2	3	4	5	6	7	8	9	10	11	12	13	14	15	16	17	18	19	20	21	22	23	24	
1. Provides accurate information about all options (IDM)	x	x		x	x	x	x		x	x		x			x	x	x	x	x	x	x	x	x	x	19
2. Helps people think about what matters to them about the options (IDM)		x		x		x	x			x		x		x								x	x	x	10
3. Supports reasoning about all options without bias (IDM)			x														x					x	x	x	5
4. Presents figures in ways to support understanding (IDM)																						x		x	2
5. Encourages people to trade-off their evaluations to make a choice (IDM)																						x		x	2
6. Encourages people to share reasoning with their health professionals (SDM)			x	x	x	x	x	x			x	x	x	x		x	x	x	x	x	x	x	x	x	19
7. Focuses thinking about the decision in the context of their lifestyle (IDM)							x																		1
8. Places the decision in the context of a changing illness-health state (IDM)	x		x	x	x	x	x	x	x	x	x	x	x	x	x	x		x	x	x	x	x		x	21
9 Enables decision to be implemented within care pathway (SDM)																							x		1
10. Encourages comparisons between different decisions (IDM)									x													x	x	x	4
Total Judgement score (out of 10)	2	2	3	4	3	4	5	2	3	3	2	5	2	3	2	3	3	3	3	3	3	7	5	8	

Resources provided risk statements about: cancer treatment side effects; cancer treatment impact on fertility; fertility preservation side effects to women and/or the baby; risk of cancer reoccurrence; success of fertility preservation treatments. Usually risk was presented as a verbal descriptor (e.g. low, high, likely) (*n* = 24), nine used percentages (SN2, SN6, SN8, SN15, SN16, SN18, SN19, SN22, SN24) and/or figures with the same common denominator across the resource (e.g. 1 in 100) (*n* = 1) (SN1), and three used graphs, bar charts and iconography figures (SN12, SN22, SN24). None provided information about the levels of uncertainty around event or outcome probabilities. Few provided balanced details about the risks and benefits of fertility preservation options; three (13%) described positive features (benefits) (SN17, SN19, SN24), and eight (33%) negative features (harms) (SN2, SN4, SN5, SN12, SN17, SN19, SN22, SN24). No resources included all the components identified for boosting informed and shared decision making [52–54]; the median score was 3 out of 10 points (range 2–8) (Table 7).

## Discussion

The search strategies identified 4851 cancer and fertility resources (Fig. 1). It took six weeks of systematic analysis to identify those integrating fertility preservation options within the cancer care pathway. Those meeting the scan’s criteria were predominantly information resources for women with cancer, raising awareness of fertility preservation and informing about infertility treatment options. Details describing the two related health problems, cancer and fertility problems, varied across resources. Three resources met the minimum criteria for recognition a patient decision aid [7, 9]; all three were developed and evaluated within research projects. Six used components and structures known to help people think actively about treatment options in accordance with their own values (Tables 4 and 7). No clinical guidelines enabled cancer professionals to prepare women for fertility preservation decisions and/or service referral; guidance was to read quality standards and/or clinical guidelines for treating fertility problems [15, 19]. As health professionals are not provided with guidance on enabling fertility preservation decisions in the context of cancer rather than infertility treatment, and relevant patient information is not easily accessible, these results explain in part why women with cancer feel simultaneously unsupported and overwhelmed by information at this time-pressured point in their treatment management [18, 22, 28, 29, 31, 73].

Using the environmental scan method provided a rigorous way to identify fertility preservation resources [42, 74]. Our data extraction sheet enabled us to critique resources systematically against established quality standards for written information [2, 4, 8, 69, 70]. However, there are limitations to using these type of web-based methods that may impact on our findings. Retrieving all resources is difficult due to the volume of material available on the internet [42, 46] and the lack of archiving and differing terminology used by developers [42]. Website content and location can change over time [75] and Google search algorithms and personalisation features linked to geographical location and previous search history all influence the results retrieved [42, 46, 75]. Ideally more than one search engine should be searched as they use different algorithms affecting relevance rankings and subsequent resource retrieval [76], patients also use different browsers or search engines. Best practice guidance for internet searches is developing [41], conducting an environmental scan using three complementary search strategies and repeating the Google search a year later from a different organisation should help to minimise the risk of missing key resources and address some of the bias in our search methods [42, 44, 74].

**Fig. 2:**
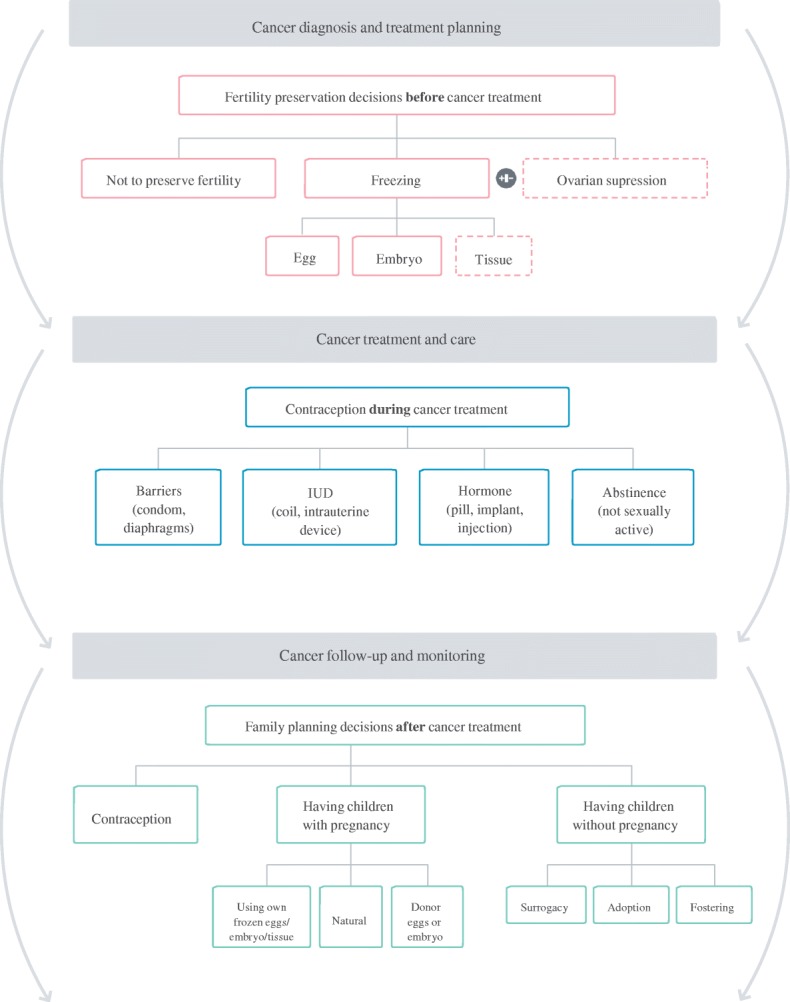
Decision map integrating fertility options within cancer-care pathway [48]

From our synthesis and critical evaluation of resources, we identified components likely to support proactively women’s health literacy and reasoning in decisions which cross medical specialty. First, explicit labelling linking the current illness with the future health problem (e.g. cancer-related fertility problem) helps establish causality between the current treatment and its iatrogenic consequence. Second, including details describing the future health across all illness representation dimensions (label/ symptoms, cause, time-line, consequences, cure/control) to enable women to have a coherent understanding of the short and long-term cancer-related fertility problems arising. Third, describing the fertility preservation decisions, and presenting all options with equivalent and balanced information, to enable stakeholders to focus on details relevant for women having cancer treatment. Fourth, signposting to other fertility-related choices within the cancer treatment trajectory raises awareness for women’s involvement at the right time in the cancer pathway [48] (Fig. 2). Fifth, describe risk figures and elicit preferences about the fertility preservation options to focus the discussion on information relevant to the context of starting, and minimising the consequences of, cancer treatment. Several resources encouraged women to rate their preferences for in-vitro fertilisation, surrogacy, adoption and fostering, i.e. options for women receiving treatment for fertility problems. People’s’ preferences are labile [77], and eliciting women’s values towards children, life-partners, and infertility treatments before they have a fertility problem, and whilst they are ill with cancer, may have limited value to making a decision about fertility preservation when being treated for cancer.

## Conclusions

Achieving patient-centred integrated cancer care requires effective communication between patients and professionals [23, 68, 78]. Current clinical guidelines, patient resources, and patient decision aid frameworks provide little guidance enabling services to provide standardised information supporting cross-specialty decisions about health options. In consequence, there is variation in what information women receive within cancer services about fertility preservation, and can access from web-sites. We suggest components based on our study’s synthesis and critical evaluation can be used in resource development guidance to inform the content and structure of patient resources, clinical guidelines and shared decision making training to support more effectively women making fertility preservation decisions before starting their cancer treatment. Providing an infrastructure to ensure adoption and maintenance of rigorously developed and evaluated patient decision aids in relevant repositories is likely to increase women’ access to appropriate resources [79]. As is raising awareness and skills of utilising social science evidence when developing and designing patient information and professional guidelines to support people making healthcare decisions.

## Additional file

Additional file 1: Environmental scan – open access patient decision aids cancer treatment_fertility preservation -- Data extraction form. (DOCX 31 kb)

## Abbreviations

AHRQ-NGC: Agency for Healthcare Research and Quality National Guidelines Clearinghouse
CFM-PtDA: Cancer Fertility and Me Patient Decision Aid
DALI: Decision Aids Library Inventory
HRA: Health Research Authority
IDM: Informed Decision-Making
IPDAS: International Patient Decision Aid Standards
NHS: National Health Service
NICE: National Institute of Health and Care Excellence
SN: Study Number of Resource
URL: Uniform Resource Location
