# Supplementary material for: Are publicly available internet resources enabling women to make informed fertility preservation decisions before starting cancer treatment: an environmental scan?
Source: BMC Med Inform Decis Mak. 2018 Nov 19;18:104. doi: 10.1186/s12911-018-0698-3 (PMC6245564; doi:10.1186/s12911-018-0698-3)
Supplement: Supplementary file 1 — Environmental scan – open access patient decision aids cancer treatment_fertility preservation -- Data extraction form. (DOCX 31 kb) [file 12911_2018_698_MOESM1_ESM.docx]

**ENVIRONMENTAL SCAN – OPEN ACCESS PATIENT DECISION AIDS CANCER TREATMENT_FERTILITY PRESERVATION**

**DATA EXTRACTION FORM.**

The data extraction form’s purpose is to enable the same type of data to be elicited from all resources, synthesised and quality evaluated using a systematic method.

From this scan, we will map the range of options being provided to women, the decisions and aspects of care supported, and the efficacy of the information to support women’s decisions. The data extraction form is informed by the International Patient Decision Aid Standards (IPDAS) Patient Decision Aid (PDA) core criteria and evidence documents, and Leventhal’s illness representation framework (AKA self-regulation theory and common sense model of illness).

The findings will inform the development of the Cancer, Fertility and Me Decision Aid (CFMDA) by illustrating what information is already provided to women to support decisions about fertility preservation options before cancer treatment, examples of good practice, and gaps in support for women.

NOTE – suggested the areas below and happy for others to be added. Piloting of data extraction will need to determine the best way of coding the data for extraction.

| **SECTION 1** | **IDENTITY DESCRIPTORS** | **CODE (TICK ALL THAT APPLY)** | **COMMENT FOR SYNTHESIS** |
| --- | --- | --- | --- |
| Study ID | ID given to PDA by CFM team |  |  |
| Name PDA | Title PDA |  |  |
| e-Location | URL |  |  |
| e-Type | Can be accessed from internet, adapted for use as internet-information and/or is internet-based. | 🞎 available (e.g. pdf, audio, posted on request)  🞎 adapted (e.g. navigation information on website)  🞎 internet-based (e.g. interactive, tailored to personal details, app, video) |  |
| Target | Who is the information for | 🞎 women adult  🞎 women teenage young adult  🞎 women under 12  🞎 parent making decisions with/for child  🞎 partner/ carer of woman with cancer  🞎 men with cancer  🞎 other |  |
| Cancer Type | What type of cancer | 🞎 breast  🞎 leukaemia  🞎 lymphoma  🞎 haematological  🞎 gynaecological  🞎 other |  |
| Authors | Names of authors | 🞎 no stated (organisation only)  🞎 lead Author |  |
| Developers (stakeholders) | Cross-sector, multi-disciplinary representations | 🞎 not stated  🞎 oncology health professionals 🞎 fertility health professionals  🞎 primary care professionals 🞎 patient and/or carer  🞎 charities 🞎 PPI advocates  🞎 professional organisation 🞎 pharma/ company  🞎 applied health researchers 🞎 App designers  🞎 decision scientists  🞎 internet-based information specialists |  |
| Year | Year published | 🞎 not stated  🞎 year |  |
| Publishers | Organisational developers | 🞎 healthcare service 🞎 charity 🞎 insurance 🞎 pharma 🞎 other |  |
| Funders | Were funders listed | 🞎 not stated  🞎 yes  🞎 conflict of interest noted |  |
| Country | Where developed | 🞎 USA 🞎 Canada 🞎 UK 🞎 NL 🞎 D 🞎Au 🞎 NZ 🞎 other |  |
| Language |  | 🞎 English 🞎 Dutch 🞎 other |  |
| Reference to other print |  | 🞎 braille 🞎 other translated version 🞎 other 🞎 not stated |  |

| **SECTION 2** | **QUALITY INDICATORS** | **CODE (TICK ALL THAT APPLY)** | **COMMENT FOR SYNTHESIS** |
| --- | --- | --- | --- |
| Endorsement I | Endorsed by a third party - patient information. | 🞎 not stated  🞎 information standard/ plain English crystal make  🞎 professional body (e.g. NICE, RCO&G)  🞎 patient body endorsement (e.g. BMA patient info)  🞎 other |  |
| Endorsement II | Endorsed by a third party – patient decision aid. | 🞎 not stated  🞎 DALI/ IPDAS  🞎 other PDA endorsement |  |
| Readability | Run text through <https://readability-score.com/>  (cut and paste 3 pages - 1 complex page, 1 simple page, 1 average page) | Average level score (e.g. Flesch)  Grade equivalent (e.g. SMOG) |  |
| Research-based | Developed systematically | 🞎 not stated  🞎 systematic development described (e.g. statement/ article)  🞎 peer-reviewed articles of development and/or evaluation |  |
| Evidence-based | Lists evidence used to inform content | 🞎 not stated  🞎 clinical effectiveness references listed  🞎 patient experience/ decision making references listed  🞎 decision science / health psychology references listed |  |
| Length | Number of pages total |  |  |
| *Other* | *Something else that good practice?* |  |  |

| **SECTION 3** | **DECISION & OPTIONS** | **CODE (TICK ALL THAT APPLY)** | **COMMENT FOR SYNTHESIS** |
| --- | --- | --- | --- |
| Purpose PDA | Stated purpose of the resource. Cut and paste intro paragraph(s) to booklet | 🞎 not stated |  |
| Health condition | Describes health condition label  identity/ symptoms  cause  time-line  consequences  cure/ control  emotional response | Cancer 🞎 Reproduction 🞎 Infertility 🞎 Cancer-related infertility 🞎  🞎 🞎 🞎 🞎  🞎 🞎 🞎 🞎  🞎 🞎 🞎 🞎  🞎 🞎 🞎 🞎  🞎 🞎 🞎 🞎  🞎 🞎 🞎 🞎 |  |
| Cancer Treatments | Describes treatments label  procedure  eligibility  cure cancer  side effects treatment  other impact | Chemo 🞎 Radio 🞎 Hormone 🞎 Surgery 🞎 OS 🞎 Other 🞎 TT 🞎  🞎 🞎 🞎 🞎 🞎 🞎 🞎  🞎 🞎 🞎 🞎 🞎 🞎 🞎  🞎 🞎 🞎 🞎 🞎 🞎 🞎  🞎 🞎 🞎 🞎 🞎 🞎 🞎  🞎 🞎 🞎 🞎 🞎 🞎 🞎 |  |
| Fertility Preservation | Describes treatments label  procedure  eligibility  cure infertility  side effects baby  side effects cancer  other impact | Egg 🞎 Embryo 🞎 OT 🞎 OS 🞎 Letrozole 🞎 Other 🞎  🞎 🞎 🞎 🞎 🞎 🞎  🞎 🞎 🞎 🞎 🞎 🞎  🞎 🞎 🞎 🞎 🞎 🞎  🞎 🞎 🞎 🞎 🞎 🞎  🞎 🞎 🞎 🞎 🞎 🞎  🞎 🞎 🞎 🞎 🞎 🞎 | Other- ovarian transposition and sparing surgery |
| Contraception | Describes need for contraception/ family planning | 🞎 during cancer treatment  🞎 For self 🞎 For partner  🞎 after cancer treatment 🞎 not stated  🞎 For self 🞎 For partner |  |
| Having a baby) | Describes fertility after cancer treatment. label  procedure  eligibility (testing, partner, etc)  cure infertility  side effects baby  side effect cancer  other impact | Natural 🞎 🞎 donor X/Y/Z 🞎 adoption 🞎 other  🞎 🞎 🞎 🞎 🞎 🞎 🞎 🞎  🞎 🞎 🞎 🞎 🞎 🞎 🞎 🞎  🞎 🞎 🞎 🞎 🞎 🞎 🞎 🞎  🞎 🞎 🞎 🞎 🞎 🞎 🞎 🞎  🞎 🞎 🞎 🞎 🞎 🞎 🞎 🞎  🞎 🞎 🞎 🞎 🞎 🞎 🞎 🞎  🞎 🞎 🞎 🞎 🞎 🞎 🞎 🞎 | X- Egg/embryo  Y- Surrogacy  Z-Sperm |
| Illness-health-QoL | Information moves through the illness-wellness trajectory and talks about quality of life / lifestyle choices (e.g. leisure, work, study) more broadly | 🞎 before cancer reproduction  🞎 during cancer reproduction  🞎 after cancer reproduction  🞎 after cancer living with infertility  🞎 not stated |  |
| Personal Circumstances | States any personal situations | 🞎 religion  🞎 ethical  🞎 relationship status  🞎 not stated |  |
| Other? |  |  |  |

| **SECTION 4** | **DECISIONAL ARCHITECTURE**  **Support active thinking**  **Minimise bias** | **CODE (TICK ALL THAT APPLY)** | **COMMENT FOR SYNTHESIS** |
| --- | --- | --- | --- |
| Decisions stated | Decisions between options are clearly stated | 🞎 no - only options / choices stated and described  🞎 between cancer treatment and FP&CT  🞎 between FP options  🞎 about having children  🞎 partially  🞎 other |  |
| Comparing between options | Options described in parallel to help comparison across options | 🞎 no described sequentially / listed  🞎 yes presented in parallel (e.g. tables/ columns)  🞎 partially – summary table of facts  🞎 other |  |
| Comparing pros/cons | Advantages and disadvantages of a choice or option compared | 🞎 no, described sequentially  🞎 yes presented in parallel (e.g. adv/ disadv in different columns)  🞎 partially – some options presented others not  🞎 other |  |
| Decision Maps | All decision and choices represented in one figure | 🞎 no  🞎 yes  🞎 partially - ‘sub’ decision (e.g. baby with/ without partner) |  |
| Metacognition | Statements to guide people in how to make decisions. | 🞎 no  🞎 partially  🞎 yes |  |
| Others values | Text includes judgments health professionals/ other patients. | 🞎 none (e.g. no value statements)  🞎 yes – health professionals (e.g. views HCP, important that…)  🞎 yes – other patients (e.g. patient story about what they chose)  🞎 yes – other patients (e.g. patient story about how reasoned)  🞎 other |  |

| **SECTION 5** | **RISK / VALUE CLARIFICATION** | **CODE (TICK ALL THAT APPLY)** | **COMMENT FOR SYNTHESIS** |
| --- | --- | --- | --- |
| Chance/ risk | Used risk figures/ statements | 🞎 none  🞎 success cancer treatment  🞎 side effects cancer treatment  🞎 infertility rates (population)  🞎 impact cancer treatment on infertility  🞎 side effects fertility preservation to woman (e.g. health/ cancer)  🞎 side effects infertility treatment to woman (e.g. cancer coming back)  🞎 side effects infertility treatment to baby  🞎 success fertility treatments |  |
| Contentious information | Misleading/out of date information or statements | 🞎 yes 🞎 partially  🞎 no |  |
| Presentation risk | How risk presented | 🞎 none  🞎 value-based descriptors (low, high, etc)  🞎 percentages  🞎 1 in XX - different denominators throughout  🞎 1 in YY – same denominators throughout  🞎 use icongraphs  🞎 use bar charts/ histograms  🞎 used both positive and negative frames (e.g. 1 /99; 98/ 99)  🞎 used other risk figures to put in context (e.g. radioactivity of x) |  |
| Value guidance | Statements to help patients think what important | 🞎 no  🞎 partially  🞎 yes |  |
| Lifestyle | QoL statements to put decision in context their lifestyle | 🞎 no  🞎 partially  🞎 yes |  |
| How using story | Patient narrative to focus on feelings and values | 🞎 no  🞎 partially  🞎 yes |  |
| Trade-off | Prompt to help trade-off ratings (e.g. balance sheet) | 🞎 no  🞎 partially  🞎 yes (e.g. trade-off question/ balance sheet/ prioritise) |  |
| Decision preference | Includes a prompt to rate preference | 🞎 no  🞎 partially  🞎 yes (e.g. leaning towards x; rank order; rate confidence choice) |  |
| **SECTION 6** | **ENGAGEMENT HEALTH SERVICE** | **CODE (TICK ALL THAT APPLY)** | **COMMENT FOR SYNTHESIS** |
| Prompts SDM | Questions to prepare for consultations | 🞎 no  🞎 yes – as headings to structure information  🞎 yes – as questions to ask health professionals  🞎 other |  |
| Prompts Friends | Questions to prepare for talking with friends/ family | 🞎 no  🞎 yes – as headings to structure information  🞎 yes – as questions to talk with friends/ family  🞎 other |  |
| Pictures | Pictures, figures to prepare for procedures | 🞎 no  🞎 yes – women’s reproductive system/ body  🞎 yes – treatments (fertility preservation)  🞎 yes – treatments (cancer)  🞎 yes – treatments (fertility – get pregnant)  🞎 other |  |
| Photos | Photographs of women, couples, health professionals, children/babies | 🞎 yes  🞎 partially  🞎 no |  |
| Stories | Women’s stories to engage with healthcare | 🞎 no  🞎 partially  🞎 yes |  |
| Signposting | Suggest people look elsewhere, provide other resources | 🞎 no  🞎 partially  🞎 yes |  |
| Signposting | Suggest people to talk to other people | 🞎 no  🞎 yes- partner  🞎 yes- family  🞎 yes- friends  🞎 yes- oncology professionals  🞎 yes- fertility health professionals  🞎 others  🞎 partially |  |

| **SECTION 7** | **Web engagement** | **CODE (TICK ALL THAT APPLY)** | **COMMENT FOR SYNTHESIS** |
| --- | --- | --- | --- |
| Not Applicable | Not an active website | 🞎 eInformation, i.e. not an interactive website or app |  |
| Navigation | Does the web-site make it clear how to navigate through the information? | 🞎 no  🞎 yes |  |
| Personalised | Does the web-site let women choose how to search for information/ navigate the website? | 🞎 no  🞎 yes |  |
| Tailored | Does the web-site ask for women’s health details to provide tailored or person-specific information? | 🞎 no  🞎 yes |  |
| Orientation | Does the web-site help women to return to the decision aid when searched for more information? | 🞎 no  🞎 yes |  |
| Tracking | Does the web-site give women feedback about what they looked at? | 🞎 no  🞎 yes |  |
| Prompts | Does the web-site have value prompts/ rating scales to provide feedback on personal preferences? | 🞎 no  🞎 yes |  |
| Drilling down | Does the web-site have information that you need to drill down for? | 🞎 no  🞎 yes |  |
| PDF | Does the web-site have a generic PDF of the decision aid for people to access? | 🞎 no  🞎 yes |  |
| Healthcare | Is the website to be used by the woman on their own, or with health professional? | 🞎 on their own before seeing fertility specialist (after cancer specialist)  🞎 on their own after seeing fertility specialist  🞎 in the consultation within cancer care  🞎 in the consultation within fertility care  🞎 in the consultation within primary care  🞎 Other |  |

| **SECTION 8** | **Judgements** | **CODE (TICK ALL THAT APPLY)** | **COMMENT FOR SYNTHESIS** |
| --- | --- | --- | --- |
| Informed | Does the decision aid provide accurate information about all options? | 🞎 no  🞎 yes |  |
| Values | Does the decision aid help women think about what matters to them about the options? | 🞎 no  🞎 yes |  |
| Bias | Does the decision aid structure help women reason about the different options without bias? | 🞎 no  🞎 yes |  |
| Risk | Does the decision aid present figures in a way that people can understand? | 🞎 no  🞎 yes |  |
| Trade-off | Does the decision aid help women to trade-off their evaluations to make a choice? | 🞎 no  🞎 yes |  |
| SDM | Does the decision aid help women share their reasoning with their health professionals? | 🞎 no  🞎 yes |  |
| Context life | Does the decision aid help women think about this decision in the context of their life? | 🞎 no  🞎 yes  🞎 partially |  |
| Context health | Does the decision aid help women think about this decision in the context of their changing health state? | 🞎 no  🞎 yes – getting back to ‘normal health’ after cancer treatment  🞎 yes – getting back to ‘reproductive health’ after cancer treatment  🞎 yes – being infertile after cancer treatment |  |
| Care Pathway | Does the decision aid help women make this decision so it can be implemented within their care? | 🞎 no  🞎 yes |  |
| Comparisons | Does the decision aid help women make comparisons between different decisions? | 🞎 no  🞎 yes |  |

**Quality assessment of DAs according to the IPDASi v4 criteria**

| **Dimensions** | **Items** | **Judgements** | **Yes** | **No** |
| --- | --- | --- | --- | --- |
| Information | 1 | DA describes health condition or problem |  |  |
|  | 2 | DA explicitly states the decision that need to be considered |  |  |
|  | 3 | DA describes the options available for the decisions |  |  |
|  | 4 | DA describes the positive features (benefits/advantages) of each option |  |  |
|  | 5 | DA describes the negative features (harms, side effects, or disadvantages) of each option |  |  |
| Values | 6 | DA describes what it is like to experience the consequences of the options (physical, psychological, social) |  |  |
| Information | 7 | DA shows the negative and positive features of options in equal detail (using similar fonts, sequence, and representation of statistical information) |  |  |
| Evidence | 8 | DA provides citations to the evidence selected (e.g. references, guidelines) |  |  |
|  | 9 | DA provides a production or a publication date (e.g. year) |  |  |
|  | 10 | DA provides information about the update policy (e.g when last revised) |  |  |
|  | 11 | DA provides information about the levels of uncertainty around event or outcome probabilities |  |  |
| Disclosure | 12 | DA provides information about the funding source used for development |  |  |
